# CmPn signaling networks in the tumorigenesis of breast cancer

**DOI:** 10.3389/fendo.2022.1013892

**Published:** 2022-09-29

**Authors:** Mellisa Renteria, Ofek Belkin, David Jang, Justin Aickareth, Muaz Bhalli, Jun Zhang

**Affiliations:** Department of Molecular and Translational Medicine (MTM), Texas Tech University, Health Science Center El Paso, El Paso, TX, United States

**Keywords:** breast cancer, CmPn signaling networks, CmP signaling networks, mPR-specific PRG actions, progesterone

## Introduction

Breast cancer is the most commonly diagnosed cancer worldwide and remains the second leading cause of cancer death in the United States (1, 2). One distinct characteristic of breast cancer is that breast cancer tumorigenesis is strongly influenced by one or more sex steroid hormones, defined as hormone-sensitive/dependent cancers. It has been well described that two major female sex steroids, estrogen (E) and progesterone (PRG), are highly associated with the growth of some types of breast cancers, which usually contain two major sex steroid receptors, estrogen receptors (ERs) and progesterone receptors (PRs). Breast cancer cells derived from these hormone-sensitive breast cancers can become activated and proliferate when they are stimulated with hormones. Based on this phenomenon, certain human reproductive cancers have been labeled hormone-related cancers, including breast, endometrium, ovarian, prostatic, and testicular cancers, which may share a unique mechanism of carcinogenesis associated with sex steroids (3).

Approximately 70% of advanced breast cancers are considered to be ‘hormone responsive’ due to their expression of ERs, PRs, or both (4, 5). However, triple-negative breast cancer (TNBC), one of the most aggressive forms of breast cancer, defined by the lack of expression of the estrogen receptor (ER), classic PRG receptors (nPRs), and epidermal growth factor receptor 2 (HER2) (6), can be considered as hormone non-responsive breast cancer. TNBC is a notoriously heterogeneous disease and yet the most poorly understood (7), exhibiting different histological and molecular subtypes with varying clinical outcomes. TNBC accounts for roughly 15% of all breast cancers and exhibits the most aggressive metastatic behavior (8) with limited targeted therapies (9). Ironically, recent data demonstrated that there is still quite a significant biological and/or clinical relevance of sex steroids in TNBCs and the action of these hormones is exerted through similar molecular mechanisms (10–14). This suggests a possible existence of previously undefined sex steroid receptor-mediated signaling pathways that retain the sensitivity and vulnerability of TNBCs to endocrine hormones (15), reemphasizing that perturbed expression of sex hormone receptors and abnormally long-exposure to sex steroids should be equally evaluated for more effective preventative strategies for breast cancers (16, 17). Efforts on this aspect have thus far identified several candidate steroid receptors, including androgen receptors (ARs), novel classic ERs (ER-βs), and G-protein-coupled ERs (GPERs), in the pathobiological actions of sex hormones in TNBCs. Endocrine therapies continue to be the anchored treatment for breast cancers, though the development of endocrine resistance has become a large obstacle for this therapeutic strategy, suggesting again that there are undefined mediators and signaling pathways associated with endocrine resistance in breast cancers (18, 19). It is important to mention that steroid hormones bind either nuclear receptors or membrane receptors. However, the most studied class of steroid hormone receptors are the nuclear receptors due to their early discoveries (20). Similarly, although the term “sex hormone” in hormone-dependent/-responsive/-sensitive breast cancers can be referred to either E or PRG (14), it usually refers only to E (21, 22). ERs can be classified into nuclear receptors (ERα and ERβ) (10) and membrane receptors (mERs) (23, 24). mERs include G protein-coupled ER1 (GPER)/G protein-coupled receptor 30 (GPR30) (11), ERx (a type of less known mERs) (25), and Gq-coupled membrane ER (Gq-mER) (26). Current hormonal therapies for breast cancer, also known as antiestrogen therapies, usually target various types of ERs and selective ER modulators (SERMs) (27, 28) by default, for the treatment and prevention of breast cancers (29, 30), demonstrating the limitation of current endocrine therapy. The effects of PRG and its corresponding receptors on breast cancers are less emphasized (31).

## CmPn/CmP signaling networks

### PRG-mediated signaling through non-classic membrane PRG receptors (mPRs)

As a sex steroid, PRG is essential for normal breast development through its positive role in promoting proliferation of human mammary epithelial cells. Therefore, PRG and its derivatives have been long suspected to be culprits for the development of breast cancers (17, 31–35). However, the underlying mechanism of how PRG plays a role in breast cancers through either enhancing EMT (epithelial-mesenchymal transition) (25, 36–40), inhibiting EMT (41), or having no effect on EMT (39), remains to be determined. Like ERs, PRG receptors can be classified into classic nuclear receptors (nPRs) and non-classic mPRs (42–46). It was reported that PRG excerpts its cellular actions through signaling cascades that involve either nPRs, mPRs, or combined responses (47). PRG binds to nPRs as a transcription factor to evoke classic actions (48). Alternatively, PRG can also bind to and activate mPRs, which subsequently leads to activation of nPRs, leading to a hypothesized model where PRG-dependent mPRs contribute to later nPR-mediated PRG actions (49). Two groups of non-classic mPRs have been recently identified, known as membrane PRG receptor/Class II progestin and adipoQ receptors (mPRs/PAQRs) and the cytochrome-related sigma-2/PRG receptor membrane components (S2R/PRGMCs) (42, 43). mPRs are highly expressed in reproductive tissues and were reported to execute rapid, non-genomic actions through interactions with G-protein coupled receptors (42, 43, 50).

### Simultaneous PRG actions can be mediated through nPRs and mPRs in parallel

Despite its significance, the relationship between nPRs and mPRs have been minimally explored. Our group recently provided strong evidence that the CCM signaling complex (CSC) plays an essential role to bridge the crosstalk between nPRs, mPRs, and their shared ligands (progestins/anti-progestins), such as PRG, to establish and modulate this cascade among nPR positive (+) breast cancer cells. There has been supporting evidence that PRG promotes cellular proliferation (51) and inhibits apoptosis in human nPR(+) breast cancer T47D cells, suggesting PRG might not be the benign hormone for nPR(+) breast cancers and may rather be pro-oncogenic (52). It has been speculated that PRG, its cellular metabolites, and its derivatives (progestins) promote cellular proliferation through induced activation of MAPK signaling pathways in both nPR(+/-) breast cancer cells, which is independent of PRs and ERs (53). These results also suggest existence of both independent and coordinated relationships between nPR-/mPR-mediated PRG signaling involved in proliferative signaling from multiple experiments with varying approaches (32, 34, 45, 51, 54–57).

### mPR-specific PRG actions

Since both types of PRG receptors and their modulators can be predominant targets for breast cancer therapy (58), as an antiprogestin (36, 59–63) [one of the well-known common contraceptives (60–65)], mifepristone (MIF, RU486, antiprogestin) has certainly earned its candidacy in the treatment of reproductive cancers (58, 66–68) such as breast, prostate, ovarian, and endometrial cancers, and has been previously studied in many clinical trials (59, 62, 63, 69). It was demonstrated that elevated levels of MIF can enhance the growth inhibition and induction of apoptosis triggered by high doses of PRG in nPR(+/-) cancer cells (70, 71). A clinically relevant dose of MIF significantly improved the treatment efficacy of chemotherapy regimens for human ovarian carcinoma cells (72). However, there have been many contradictory results reported regarding whether MIF has growth inhibition or stimulation for hormone-responsive breast cancer cells, as an anti-progestin (73). MIF can act as a potent antagonist of steroid hormone receptors such as nuclear PRG receptors (nPRs), glucocorticoid receptors (GRs), and androgen receptors (ARs) through directly binding as the ligand (74–78). The degree of nPRs, GRs, and ARs inhibition by MIF are variable, depending on dosages of MIF and specific cell types (79). Recent data demonstrated that only nPRs play protective roles on the stability of the CSC under the negative effects of mPR-specific PRG actions, while both GRs and ARs play no role in this CmPn signaling network (57). Among three types of antiprogestins, MIF is defined as a type-II antiprogestin, which can also act as an agonist in nPR(+) tissues in a cell-specific manner, based on selective modulation of PRs (SPRM) criteria (80). MIF has been well known to exert its antagonist action, *in-trans*, with three dimeric forms of nPR isoforms (AA, AB, and BB) to inhibit nPR activation at concentration that are substoichiometric with PRG (81, 82), however, it is also reported that MIF can affect the ratio of nPG-A/nPG-B isoforms at both the transcriptional and translational levels, depending on dosages of MIF and specific cell types (83). Since MIF can act as an agonist, and the patient survival outcome depends on the ratio of nPR antagonists versus agonists effects of MIF, the mechanism of how MIF is either nPR antagonists or agonists needs to be define. In an *in-vitro* experiment with nPR knockout T47D cells (T47D-Y), cellular expression transactivation was observed only in the T47D-Y cells ectopically expressed nPR-B isoform (T47D-YB) (84), suggesting that nPR-B isoform has a unique activation domain that may confer agonist-like properties in the presence of MIF (85). Recent findings demonstrated that the regulated expression of nPRA and nPRB is critical to the breast cells’ response to synthetic progestins, this altered PRG receptor expression may be an important factor in the malignant transformation of breast cells (86). Regardless of tissue of origin and hormone responsiveness, the anti-proliferative activity of MIF in cancer cells has been found to be independent of nPRs (87). Similarly, the cellular effects of MIF on proliferation were also reported (54, 88). It seems that the pro- or anti-proliferative activities of MIF are cell-specific (69) and might be determined by the applied dosage of MIF, as well as ratio of nPR isoforms (36). These results indicate the importance to uncover key mediators of MIF and underlying mechanisms of its dual roles (pro- or anti-tumor activity) in breast malignancies (87). The possible convergence of classic and non-classic PRG actions (nPRs/mPRs) and CSC signaling on their common cellular targets in nPR(+) cells is an attractive model by which PRG and MIF can fine tune this intricate balance among these signaling pathways. Our recent findings suggest that combined PRG and MIF treatments can enhance the inhibitory effects on protein expression of CSC, which is independent of nPR(+/-) status. This indicates that PRG and MIF can work synergistically to inhibit the protein stability of the CSC, only through mPRs, termed as mPR-specific PRG action (57, 89–92).

### CSC-mPRs-PRG-nPRs (CmPn) signaling network in nPR(+) breast cancer cells

Altered expression patterns of *nPRs*, *mPRs* and *CCM* genes across clinical breast tumors and their associated prognostic effects were observed (93), suggesting combined involvement of all three signaling pathways in influencing the size and extent of the primary breast tumor (57, 90, 91). Our recent findings that upon treatment of PRG and MIF, which only excerpt their PRG actions through mPR-specific PRG actions, the expression patterns of three CCM proteins which form the CSC are altered in the nPR(+) breast cancer cells, T47D, strongly suggesting the involvement of the CSC in breast tumorigenesis (57, 90, 91). Reciprocally, mPRs and nPRs signaling cascades are coupled through CSC modulation (57). These results solidify a novel network among the CSC, classic, and non-classic PRG receptors, termed the CSC-mPRs-PRG-nPRs (CmPn) signaling network, which is dynamically modulated and fine-tuned with a series of feedback regulations under PRG actions.

### CSC-mPRs-PRG (CmP) signaling network in nPR(-) breast cancer cells

**Figure 1:**
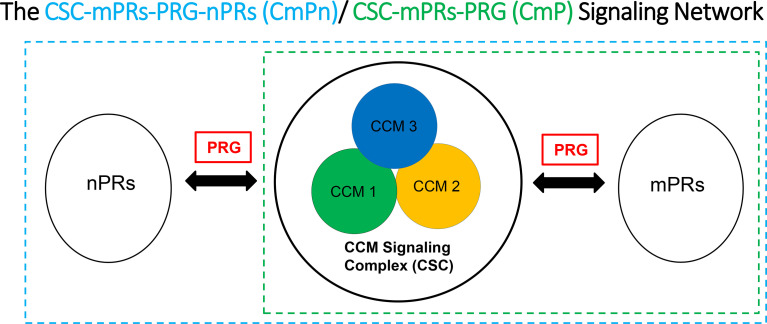
Signaling network for key CSC players and the effects of progesterone (PRG) on nPR and mPR. We suggest that the CSC functions as a channel modulator for the interactions of PRG between nPRs and mPRs.

Strong evidence for the existence of PRG-mPRs signaling cascade in both nPR(+/-) breast cancer cells have been previously proposed (41, 94–97), suggesting that PRG signaling in nPR(-) cell lines can be mediated solely through mPR-mediated signaling (termed as mPR-specific PRG actions) (41). In our recent reports, we further defined the novel CSC-mPRs-PRG (CmP) signaling network in nPR(-) breast cancer cells which overlaps with our previously defined CmPn (CSC-mPRs-PRG-nPRs) network in nPR(+) breast cancer cells (57). In the CmP signaling network, the CSC is able to stabilize mPRs under steroid actions in a forward fashion (CSC➔mPRs), indicating an essential role of the CSC in maintaining the stability of mPRs in nPR(-) breast cancer cells under the mPR-specific PRG actions. Overall, the CSC can stabilize the expression of mPR proteins in TNBC cells in concordance with our observations in nPR(+) breast cancer cells, indicating the consistent function of the CSC on the stability of mPRs under PRG actions (57). Since nPR-mediated signaling pathways usually have protective roles over the CSC, the intricate balance of the CmPn signaling network is more stable under mPR-specific PRG actions compared to the CmP network. Therefore, in contrast to our previously observed nPR(+) breast cancer and nPR(-) endothelial cell data (57, 89, 92), mPR-specific PRG actions have variable effects on both RNA and protein expression levels on the CSC in TNBCs, which can be utilized for subtype classifications (90, 91). In general, the relationships among the CSC, mPRs, PRG, nPRs, and their mediated signaling pathways within either the CmPn or the CmP signaling networks can be summarized (**Figure 1**). Extensive omics has been performed confirming alterations in key tumorigenesis signaling pathways, suggesting their association with the CmPn/CmP signaling network in nPR(+/-) breast cancers (57, 90, 91, 93, 98).

## Discussion

The interplays between classic and non-classic PRG-mediated signaling have been long suspected (43, 44, 49, 99) and newly discovered evidence demonstrated that the CSC plays an essential role to bridge crosstalk among nPRs, mPRs, and their ligands to form the CmPn/CmP signaling networks in response to mPR-specific PRG actions among nPR(+/-) breast cancer cells (57, 90, 91, 93). The convergence of classic and non-classic PRG actions by the CSC on their common ligands and downstream cellular targets in breast cancer cells is an attractive model by which mPR-specific PRG actions play a central role for the stability of this system by fine-tuning the intricate balance within CmPn/CmP signaling network. Therefore, any activities that disrupt this intricate balance within the network, including patients under hormone replacement therapy (HRT), females taking hormonal contraceptives, or extended exposures (dietary and/or daily supplementation) to hormones during their reproductive ages, could result in perturbation of the CmPn/CmP signaling networks, with potential serious consequences of increased risks in breast cancers and compromised tumor therapy.

## Author contributions

JZ proposed, designed and drafted manuscript. MR, OB involved in revision and comments. DJ, JA and MB involved in comments. All authors contributed to the article and approved the submitted version.

## Conflict of interest

The authors declare that the research was conducted in the absence of any commercial or financial relationships that could be construed as a potential conflict of interest.

## Publisher’s note

All claims expressed in this article are solely those of the authors and do not necessarily represent those of their affiliated organizations, or those of the publisher, the editors and the reviewers. Any product that may be evaluated in this article, or claim that may be made by its manufacturer, is not guaranteed or endorsed by the publisher.
